# Breast cancer supplemental screening: Women’s knowledge and utilization in the era of dense breast legislation

**DOI:** 10.1002/cam4.3218

**Published:** 2020-06-14

**Authors:** Jenerius A. Aminawung, Jessica R. Hoag, Kelly A. Kyanko, Xiao Xu, Ilana B. Richman, Susan H. Busch, Cary P. Gross

**Affiliations:** ^1^ Cancer Outcomes, Public Policy, and Effectiveness Research (COPPER) Center Yale School of Medicine New Haven CT USA; ^2^ Department of General Internal Medicine Yale University School of Medicine New Haven CT USA; ^3^ Department of Population Health New York University School of Medicine New York City NY USA; ^4^ Department of Obstetrics, Gynecology and Reproductive Sciences Yale University School of Medicine New Haven CT USA; ^5^ Department of Health Policy and Management Yale School of Public Health New Haven CT USA

**Keywords:** breast cancer screening behavior, breast density, dense breast notification laws, patient provider discussion, supplemental screening

## Abstract

**Background:**

Given the growth in dense breast notification (DBN) legislation in the United States, we examined the association between different types of DBN laws and supplemental screening behaviors among women.

**Methods:**

We surveyed in March–April 2018 a nationally representative sample of women aged 40‐59 years who received a routine screening mammogram in the past 18 months. Survey items included the following topics regarding supplemental screening: discussing risks or benefits with a provider, knowledge about the risk of false positives, and utilization. We grouped women by state DBN into non‐DBN, generic DBN (mentions breast density but not supplemental screening), DBN that mentions supplemental screening (DBN‐SS), and DBN with mandated insurance coverage for supplemental screening (DBN‐coverage), and estimated adjusted predicted probabilities for supplemental screening behaviors.

**Results:**

Of 1641 women surveyed, 21.3% resided in non‐DBN, 41.2% in generic DBN, 25.8% in DBN‐SS, and 12.5% in DBN‐coverage states. Overall, 23.0% of respondents had discussed supplemental screening with a provider, 11.3% of whom discussed the risks, and 49.5% discussed the benefits. In adjusted analysis, women living in DBN‐coverage states were more likely to discuss supplemental screening (27.5%) than women in non‐DBN states (13.6%); pairwise contrast 13.8% (95% CI, 2.1% to 25.6%; *P *= .01). They were also more likely to have received supplemental screening for increased breast density (19.3%) compared to women living in non‐DBN (9.9%); contrast 9.4% (95% CI, 1.6% to 17.3%; *P *= .01), Generic DBN (7.3%); difference 12.0% (95% CI, 4.6% to 19.4%; *P* =< .001), and DBN‐SS (8.8%); contrast 10.5% (95% CI, 2.6% to 18.5%; *P *< .01) states.

**Conclusions:**

Women in DBN‐coverage states were more likely to discuss supplemental screening with their providers, and to undergo supplemental screening, compared to women in states with other types of DBN laws, or without DBN laws.

## Graphical abstract

In a nationally representative survey, women living in states with dense breast notification laws requiring insurance coverage for supplemental screening in the context of increased breast density were more likely to have discussed supplemental screening with a provider, and to have received supplemental screening. There was a limited knowledge of risks associated with supplemental screening, which underscores the need for increased awareness of risks and benefits, as well as the need to generate additional evidence regarding effectiveness of supplemental screening for women with increased breast density.

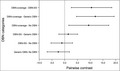

## 1 INTRODUCTION

In 2009, Connecticut became the first state to implement dense breast notification (DBN) legislation, requiring mammogram reports to include language informing women about the limited sensitivity of mammography in the setting of dense breasts.^1^ Driven primarily by grassroots patient advocacy organizations,^2^ the DBN law in Connecticut was the first in a decade‐long wave of legislative efforts.^3^ By March of 2019, 37 state legislatures and the United States (US) congress had passed legislation that required DBN, and 3 states had efforts for education or reporting that do not require notification.^4^ The US Food and Drug Administration (FDA) in March 2019 announced a proposed new rule to the Mammography Quality Standard that will require DBN.^5^

There is marked variation in the content and approach of DBN laws across states.^6^ In addition to highlighting the “masking effect” of dense breasts on mammography, some DBN language mentions the potential benefits of supplemental screening with ultrasound or MRI, thereby encouraging women to pursue supplemental testing.^7^ Three states (Connecticut, New York, New Jersey) further mandate insurance coverage for supplemental screening ultrasound and/or MRI for women with dense breasts.

The upsurge in DBN legislation has occurred in the absence of evidence demonstrating that supplemental screening improves clinical outcomes.^8^ Though the ACRIN 6666 trial found that the addition of ultrasound and MRI to mammography for higher risk women with dense breasts detects additional cancers,^9^ the US Preventative Services Task Force, in a 2016 update to its breast cancer screening recommendations, concluded that the current evidence is inconclusive to recommend supplemental screening in women with dense breasts who have a negative screening mammogram.^10^ A recent study found that adding screening ultrasound to mammography was associated with similar cancer detection and interval cancer rates, but more than double the risk of a false‐positive biopsy.^11^ In a cost‐effectiveness analysis, supplemental screening with ultrasound among women with dense breasts was found to substantially increase costs while producing limited health benefit, either in the number of breast cancer deaths averted or in the quality‐adjusted life‐years gained.^12^

Further understanding of the association between DBN legislation and patient knowledge and behavior is particularly timely. First, the effort has evolved from a state specific to a national one: recently proposed updates to FDA mammography regulations include specific language regarding breast density be conveyed to patients.^5^ Secondly, both the state and national efforts aim to promote the delivery of information to patients about additional medical tests at a time when evidence is still under development.^13^ Hence, it is important to carefully assess the impact of DBN legislation, including whether DBN laws are associated with changes in knowledge or use of supplemental screening, and whether these associations vary with DBN legislation language. Prior work using payer claims has suggested that implementation of DBN laws was associated with a modest increase in utilization of supplemental screening with breast ultrasound and MRI.^14^, ^15^, ^16^, ^17^, ^18^ A recent survey of women aged 40‐74 found that women residing in states with DBN reported higher rates of patient–provider discussions regarding supplemental screening.^19^ However, these studies were restricted to patients with specific insurance types, and neither explored variation across states in DBN language, whether women discussed risk or benefits, nor whether they received supplemental screening. To address these gaps, we conducted a nationally representative survey to examine the association of different types of DBN with reported discussions with providers, knowledge, and utilization of supplemental screening among women aged 40‐59 years, for whom the prevalence of breast density is the highest.^20^

## 2 METHODS

### 2.1 Data and study population

We conducted a national survey through the Growth from Knowledge (GfK) KnowledgePanel^®^, a probability‐based panel of 55 000 households that is representative of the US population.^21^, ^22^, ^23^, ^24^ GfK recruits panel members using address‐based sampling methodologies, and households without Internet connection are provided with a web‐enabled device and free Internet service. Panel members provide consent and receive modest incentives to encourage participation in surveys, including raffles for cash and other prizes. We constructed and tested our survey through 12 cognitive interviews to ensure understandability of question wording and eliminate questions that could not be reliably answered by self‐report.^25^ GfK fielded the survey based on an equal probability selection method between March and April 2018 to non‐institutionalized English‐speaking women aged 40‐59 years living in the US (excluding Alaska and Washington, DC). During the field period, GfK sampled 10 112 panelists, of which 6896 completed the screening questions, resulting in a survey completion rate of 68.2% (excluding breakoffs) using the standard definition for probability‐based Internet panels.^26^ After excluding women who had never received a screening mammogram in their lifetime and those who had a history of breast cancer, 2539 (36.8%) of respondents qualified for our study.

**FIGURE 1:**
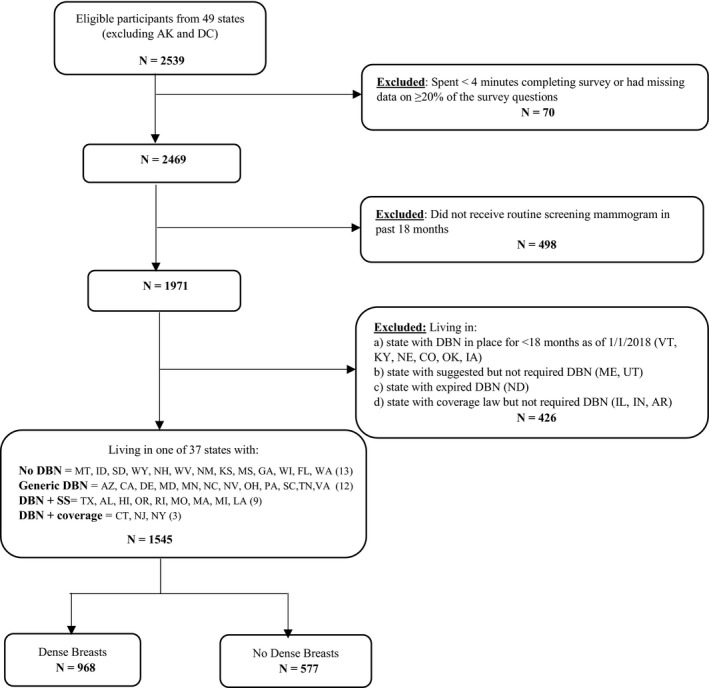
Sample selection CONSORT diagram. Counts are unweighted

Of the 2539 women who qualified for the study, we excluded 70 (2.8%) respondents who spent fewer than four minutes completing the survey (below 5th percentile of survey completion time) or had missing data on ≥ 20% of the survey questions to eliminate potential data quality concerns. Those excluded were more likely to be Hispanic and have lower education and income. We further restricted the sample to those that received a screening mammogram in the past 18 months and after DBN implementation, excluding 498 respondents who had not received a screening mammogram in last 18 months; and 426 from states with DBN laws in place for less than 18 months as of January 1, 2018 (VT, KY, NE, CO, OK, IA). We also excluded participants from states with suggested, but not required, DBN laws (ME, UT), states with expired DBN laws (ND), or states with coverage laws without required DBN (IL, IN, AR), resulting in a final sample size of 1545 (Figure 1 **)**. All survey responses were deidentified and weighted to be representative of the female US population aged 40‐59 years based on demographic benchmarks from the most recent American Community Survey using age, race/ethnicity, census region, education, and household income. Survey weights also accounted for oversampling of women with dense breasts, based on responses to the survey question on whether they had been told they had dense breast (see Appendix A).

### 2.2 Measures

GfK collected panel members’ self‐reported sociodemographic information including age, race/ethnicity, education, metropolitan statistical area status, marital status, employment status, household income, type of insurance and state of residence. The first two authors manually assigned states based on the content of their publicly available DBN legislation language,^2^, ^4^ and supplemented by the states’ legislative websites into four groups: (a) non‐DBN (no reported DBN law); (b) generic DBN (DBN language mentions breast density but not supplemental screening); (c) DBN‐SS (DBN language mentions supplemental screening); and (d) DBN‐coverage (DBN with language mandating insurance coverage for supplemental screening) (Table 1). Disagreements were resolved through consensus discussion with the senior author. We asked respondents if they had been informed by a healthcare provider or mammogram letter that they had dense breasts as well as their knowledge and behaviors related to supplemental screening.

**TABLE 1:** Dense breast notification categories, sample language, and list of states

DBN category	Sample language	States
Non‐DBN	No legislative language	FL, GA, ID, KS, MS, MT, NH, NM, SD, WA, WI, WV, WY
Generic DBN	"Your mammogram demonstrates that you have dense breast tissue, which could hide abnormalities. Dense breast tissue, in and of itself, is a relatively common condition. Therefore, this information is not provided to cause undue concern; rather, it is to raise your awareness and promote discussion with your health care provider regarding the presence of dense breast tissue in addition to other risk factors."	AZ, CA, DE, MD, MN, NC, NV, OH, PA, SC, TN, VA
DBN‐SS	"Your mammogram indicates that you have dense breast tissue. Dense breast tissue is relatively common and is found in about forty percent (40%) of women. The presence of dense tissue can make it more difficult to detect cancers in the breast by mammography because it can hide small abnormalities and may be associated with an increased risk. Hence, you may benefit from supplementary screening tests, which may include a breast ultrasound screening, or a breast MRI examination, or both, depending on your individual risk factors.”	AL, HI, LA, MA, MI, MO, OR, RI, TX
DBN‐coverage	"Screening and diagnostic imaging for the detection of breast cancer, including diagnostic mammograms, breast ultrasounds, or magnetic resonance imaging, covered under the policy shall not be subject to annual deductibles or coinsurance."	CT, NJ, NY

Abbreviations: DBN, Dense breast notification; DBN‐coverage, Dense breast notification with insurance coverage for supplemental screening; DBN‐SS, Dense breast notification that mentions supplemental screening.

We asked respondents if they had discussed supplemental screening with their healthcare provider in the past 18 months and the content of those discussions in terms of risks and benefits, to assess patient involvement in breast cancer screening decisions. We included text to distinguish screening from diagnostic tests, and defined supplemental screening as a test to look for spots or suspicious findings in women who do not have any worrisome signs or symptoms of breast cancer (see Appendix). We also asked women whether their provider had encouraged them to undergo supplemental screening. To appraise women's knowledge related to the risks of supplemental screening, we asked respondents about false positives associated with supplemental screening. Specifically, we had respondents estimate the relative number of biopsies required that turn out not to be cancer (“fewer”, “the same number”, or “more”) if supplemental screening was added to mammography. We categorized responses as correct if “more” was selected. Finally, we asked respondents if they had received supplemental screening breast ultrasound or MRI tests in the past 18 months.

### 2.3 Statistical analysis

We compared sociodemographic characteristics across the four DBN categories using Chi‐squared tests. Our results are reported as unweighted counts and weighted percentages in unadjusted analysis. We estimated multivariate logistic regression models with cluster‐robust standard errors (clustered on state of residence) to examine the association between DBN status and reported participant–provider discussion about supplemental screening (including whether such discussion occurred and risks and benefits of supplemental screening were discussed); provider encouragement to undergo supplemental screening; knowledge about the risks of supplemental screening; and utilization of supplemental screening because a screening mammogram result showed increased breast density. We also assessed the association between DBN status and reported history of breast density. In our primary models we used all four categories of DBN (Non‐DBN; generic DBN; DBN‐SS; DBN‐coverage). We adjusted for respondents’ sociodemographic characteristics in all models. We assessed whether the effect of DBN laws on supplemental screening was mediated by patient–provider discussion using the Baron and Kenny's method for categorical mediator and dependent variables.^27^ The magnitude of the relation between DBN laws and reported receipt of supplemental screening was unchanged and remained significant after adding reported patient‐provider discussion in the model thus we included patient–provider discussion and self‐reported increased breast density in our final model assessing the association between DBN and utilization of supplemental screening. We calculated adjusted predicted probabilities for each model's outcome, using prediction at the means method that assumes every person has the mean value for each covariate in the model. All statistical tests were two‐sided, with a value of *P* < .05 considered statistically significant and the Bonferroni correction applied for pairwise comparisons in adjusted analysis. We performed analyses using Stata version 14.1 (Stata Corporation), and SPSS Version 24.0. (IBM Corp.). The study protocol was approved by the Yale University Human Investigation Committee.

## 3 RESULTS

### 3.1 Survey participants

Of the 1545 women who reported receiving a screening mammogram in the past 18 months, median age was 51 years, 62% were white, 13% black, and 15% Hispanic. With respect to the four DBN categories, 21.3% of respondents were living in non‐DBN states, 41.2% in generic DBN states, 25.8% in DBN‐SS states, and 12.5% in DBN‐coverage states. Almost half (47.1%) of all respondents had been informed by a healthcare provider that they had dense breasts. Women living in DBN‐coverage states were more likely to report increased breast density (56.0%) compared to those in non‐DBN states (37.8%), Generic DBN (48.1%), and DBN‐SS (49.2%) (all pairwise comparisons vs DBN‐coverage: *P *< .001; Table 2).

**TABLE 2:** Participant sociodemographic characteristics by state DBN law

	All women (N = 1641)	Non‐DBN (n = 349)	Generic DBN (n = 676)	DBN—SS (n = 423)	DBN—Coverage (n = 193)	*P*‐value
	%	%	%	%	%	
Age						
40‐49	43.6	42.1	44.4	44.7	41.5	.79
50‐59	56.4	57.9	55.6	55.3	58.5	
Race/Ethnicity						
White non‐Hispanic	62.6	63.9	65.4	59.6	56.8	<.001
Black non‐Hispanic	13.4	16.3	7.7	20.6	12.5	
Hispanic	15.0	14.6	13.8	13.0	24.5	
Other non‐Hispanic	9.0	5.2	13.2	6.9	6.3	
Education						
Less than high school	8.5	7.7	7.8	7.8	14.0	.03
High school	23.5	22.6	20.8	26.2	28.5	
Some college	29.7	30.9	31.3	27.7	25.9	
Bachelor's degree or higher	38.3	38.7	40.0	38.3	31.6	
Household income						
<$12500	5.9	7.4	4.0	7.1	7.2	.001
$12500 to $49999	20.6	21.7	18.9	22.2	21.1	
$50000 to $99999	29.8	32.6	32.7	28.4	18.0	
>=$100000	43.6	38.3	44.4	42.3	53.6	
Employment status						
Not employed	29.4	27.7	28.6	34.3	25.4	.07
Employed	70.6	72.3	71.4	65.7	74.6	
Marital status						
Never married	10.8	12.3	8.7	11.6	14.0	<.001
Married	68.5	64.9	73.4	70.2	53.9	
Other^a^	20.7	22.9	17.9	18.2	32.1	
Insurance						
Employer sponsored	69.9	66.1	71.7	73.0	68.6	<.001
Medicare	5.5	6.0	5.5	6.4	2.6	
Medicaid	9.5	10.1	7.3	7.6	20.9	
Other^b^	11.0	12.4	12.4	10.2	5.8	
None	3.4	5.5	3.0	2.8	2.1	
Neighborhood status						
Non‐metro	10.0	14.6	7.7	11.1	6.8	<.001
Metro	90.0	85.4	92.3	88.9	93.2	
Reported dense breast						
No	52.9	62.2	51.9	50.8	44.0	<.001
Yes	47.1	37.8	48.1	49.2	56.0	

Note

Values may not sum to exactly 100% due to rounding of weighted survey responses.

Abbreviations: DBN, Dense breast notification; DBN‐SS, Dense breast notification that mentions supplemental screening; DBN‐coverage, Dense breast notification with insurance coverage for supplemental screening.

^a^ Widowed, divorced, separated.

^b^ State or federal health Insurance marketplace; Veteran's Affairs (VA), Department of Defense, or other military programs; some other source.

### 3.2 Patient–provider discussion about supplemental screening

**FIGURE 2:**
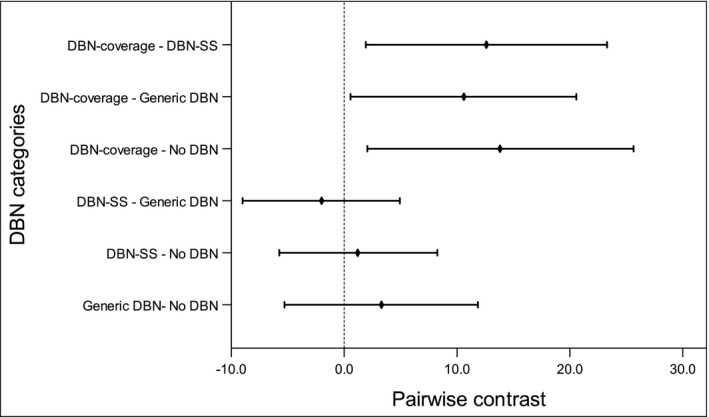
Pairwise contrast for adjusted predicted probabilities of supplemental screening discussions with healthcare provider by state dense breast notification law

In our sample, 23.0% of all women reported that they had discussed supplemental screening with a healthcare provider in the past 18 months, with 32.5% of those who reported increased breast density and 14.5% of those who did not doing so. In adjusted analysis, those living in a DBN‐coverage state were more likely to report discussing supplemental screening with their provider (27.5%) compared to women in non‐DBN states (13.6%); pairwise contrast 13.8% (95% CI, 2.1% to 25.6%, *P* = .01), generic DBN states (16.9%); difference 10.6% (95% CI,0.6% to 20.6%, *P* = .03), and DBN‐SS states (14.9%); difference 12.6% (95% CI, 1.9% to 23.2%, *P* = .01). There were no significant differences in the adjusted percentage of women who discussed supplemental screening with their providers for other pairwise comparisons of DBN categories (Figure 2).

Among women who discussed supplemental screening with their provider, they were more likely to have discussed the benefits (49.5%) than the risks (11.3%). In adjusted analysis, women living in states with generic DBN laws were less likely to report discussing the benefits of supplemental screening with their providers (21.2%) compared to women in DBN coverage states (38.2%); difference −17% (95% CI, −32.0% to −1.5%, *P* = .02) and those in non‐DBN states (34.8%); contrast −13% (95% CI, −26.0% to −0.9%; *P* = .03). There was no significant difference between women across the four DBN categories with regard to discussion of risk of supplemental screening with providers (Table 3).

**TABLE 3:** Dense breast notification (DBN) category and reported patient–provider discussion about supplemental screening

	Women who discussed supplemental screening with their provider (N = 377; 23.0%)		All women (N = 1641)
	Discussed risks	Discussed benefits	Encouraged to undergo supplemental screening
	Adjusted Predicted Probability (%)	Adjusted Predicted Probability (%)	Adjusted Predicted Probability (%)
Categorical DBN law^a^			
Non‐DBN	7.8	34.9^*^	16.3
Generic DBN	12.4	21.5^*,‡^	12.1^¶^
DBN‐SS	8.3	26.0	11.5^†^
DBN‐coverage	6.8	38.2^‡^	26.5^†,¶^

Significant Bonferroni pairwise contrast: For discussed benefits: ^*^Non‐DBN and Generic DBN = 13% (95%CI 1% ‐ 26%; *P*=.02), ^‡^DBN‐coverage and Generic DBN = 17% (95%CI 2% ‐ 32%; *P*=.03) For encouraged to under supplemental screening: ^†^DBN‐coverage and DBN‐SS = 15% (95%CI 4% ‐ 25%; *P*=.001); ^¶^DBN‐coverage and Generic DBN = 14% (95%CI 2% ‐ 32%; *P*=.001) No statistically significant difference between the other DBN categories.

Abbreviations: DBN, Dense breast notification; DBN‐SS, Dense breast notification that mentions supplemental screening; DBN‐coverage, Dense breast notification with insurance coverage for supplemental screening.

^a^ Models adjusted for age, race, education, marital status, household income, metropolitan status of respondents’ neighborhood, employment status, insurance coverage, and breast density.

Overall, 20.4% of respondents reported they had been encouraged by a healthcare provider to undergo supplemental screening. In adjusted analysis, women living in DBN coverage states were more likely to report that they had been encouraged to undergo supplemental screening by a healthcare provider (26.5%) compared to women in Generic DBN states (12.1%); pairwise contrast 14.4% (95% CI, 4.2% to 24.5%; *P *= .001) and DBN‐SS states (11.5%); difference 14.9% (95% CI, 4.4% to 25.4%; *P *= .001). The observed difference between DBN‐coverage and non‐DBN (16.3%) was not statistically significant (Table 3). Women with dense breasts were more likely to report they had been encouraged to undergo supplemental screening (28.0%) compared to those with non‐dense breasts (8.3%).

### 3.3 Knowledge of the risks associated with supplemental screening

In all, 861 respondents (52.5%) were aware that supplemental screening increases the risk of false positives compared to mammography alone. In adjusted analysis, knowledge of increased risk of false positives was similar between women reporting dense breasts and those who did not (adjusted: 61.0% vs 59.1%, *P *= .50). Adjusted rates of knowledge also did not differ significantly across categories of DBN.

### 3.4 Utilization of supplemental screening

**FIGURE 3:**
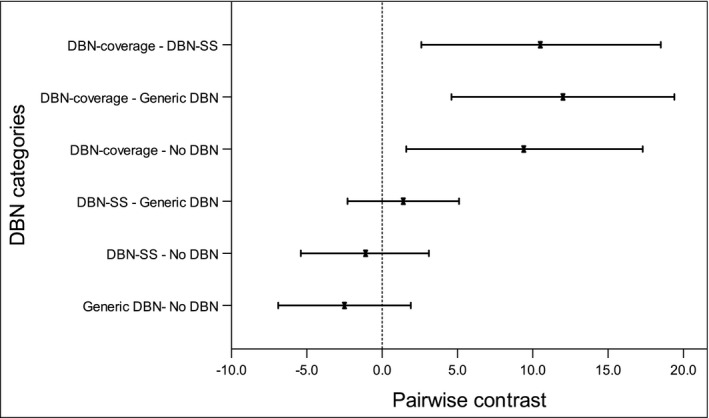
Pairwise contrast for adjusted predicted probabilities of supplemental screening utilization by state dense breast notification law

In the full sample, 20.7% of participants reported receiving supplemental screening within the past 18 months, with 4.3% receiving MRI, 18.8% an ultrasound, and 2.4% both MRI and ultrasound. About half of these women (9.3% of the total) reported receiving supplemental screening because a screening mammogram result showed increased breast density. A higher proportion of women living in DBN‐coverage states reported receiving supplemental screening (36.5%) compared to women in non‐DBN (20.6%), generic DBN (16.4%), and DBN‐SS states (20.6%, *P *< .001). In adjusted analysis, reported supplemental screening because of increased breast density was significantly higher among women residing in DBN‐coverage states (19.3%) compared to women living in other DBN categories—non‐DBN (9.9%); difference = 9.4% (95% CI, 1.6% to 17.3%; *P *= .01), Generic DBN (7.3%); difference = 12.0% (95% CI, 4.6% to 19.4%; *P *< .001), DBN‐SS (8.8%); difference = 10.5% (95% CI, 2.6% to 18.5%; *P *< .01) (Figure 3).

## 4 DISCUSSION

Our study provides nationally representative estimates of the association between ongoing state DBN legislative efforts and patient–provider discussion, knowledge, and utilization of supplemental screening. We found that women in DBN‐coverage states reported higher rates of discussion with healthcare providers about supplemental screening, more provider encouragement to receive supplemental screening, and utilization of these tests compared to women in states with non‐DBN laws, and in most instances, compared to women in states with other types of DBN.

We found that relatively few women discussed supplemental screening with their healthcare providers (23% of women indicated having such a discussion), and that generic DBN laws were not associated with higher rates of discussion. Moreover, we found that even among those who did discuss supplemental screening, only 11% discussed the associated risks. Among women reporting increased breast density, only a third of them reported discussing supplemental screening with their provider after their most recent screening mammogram. These findings suggest that not all women are benefiting from the advocated improved patient involvement in breast cancer care decisions that motivated the DBN laws.^9^ Although it is not clear why so few women reported discussing risks, it is possible that conflicting recommendations from professional medical societies and patient advocacy groups present a challenge to shared decision making between women who undergo screening mammography and their providers.^28^, ^29^, ^30^ Notably, a study of primary care providers in Massachusetts found that half of providers felt unprepared to discuss breast density or options for supplemental screening.^28^ In a similar study of California physicians, only 6% considered themselves to be “completely comfortable” answering patient questions about breast density.^29^ Contrarily, for providers in states with DBN‐coverage laws, an increased feeling of obligation to order supplemental screening may be present. In fact, a retrospective chart review following DBN‐coverage legislation in New Jersey found an increase in ultrasound requests and utilization even for patients without dense breast tissue.^16^ That we found significantly higher rates of supplemental screening utilization among women who discussed supplemental screening with their providers, and considering the fact that providers were more likely to discuss benefits than risks of supplemental screening suggest that patient–provider conversations enhance utilization.

It is striking that almost half of the women in our study had limited knowledge about the increased risk of false positives associated with supplemental screening. The contents of the DBN language may also contribute to this knowledge deficit. While many DBN laws mandate that mammography reports incorporate verbiage describing the lower sensitivity of mammograms in the setting of dense breasts, the potential benefits of supplemental screening, or encouragement to discuss these options, the risks are rarely mentioned.^6^ Provider discomfort or unpreparedness to discuss supplemental screening may also contribute to the limited knowledge on the risk of supplemental screening. A limited knowledge of the increased risk of false positives associated with supplemental screening may lead to unintended consequences as false‐positive breast cancer screening has been shown to impact screening behaviors and have long‐term psychosocial consequences in women.^31^, ^32^

We found that women in DBN‐coverage states had higher rates of supplemental screening. This finding is consistent with research suggesting that state laws requiring private insurers to cover screening mammograms play a role in increasing rates of mammography.^33^ Our observed difference in supplemental screening in DBN‐coverage states compared to other DBN categories (which were not associated with higher supplemental screening rates) is noteworthy. First, it highlights the possibility of disparities to access as some women may not be able to pay for supplemental screening in states where the test is not fully covered. Secondly, it underscores the need to revisit the language of DBN laws if the goal is to improve breast cancer outcomes. Our findings of no significant difference in supplemental screening between Generic DBN, DBN‐SS, and Non‐DBN states are similar to prior findings that noted no significant difference in pre‐ and post‐supplemental screening rates following DBN in states that mention supplemental screening only suggested but do not mandate coverage.^15^ Other studies have however noted an increase in supplemental screening in states with DBN laws.^18^ A claims‐based analysis found that women living in states with DBN‐SS were more likely to receive supplemental screening than women living in generic DBN and non‐DBN states.^17^

### 4.1 Limitations

Our study was cross sectional and cannot capture prior patient–provider discussions that may have influenced screening decisions during the study period nor can we determine whether rates of supplemental screening changed in these states after the law was implemented, as previous studies have suggested.^14^, ^15^, ^16^ We only surveyed women aged 40‐59, thus our findings may not apply to other ages in whom breast cancer screening is recommended. We assigned participants to their state of residence and not the state in which they received breast cancer screening, and some of the difference observed in our study could result from variation in healthcare delivery across states. Our survey relied on self‐reported responses about supplemental screening‐related behavior in the past 18 months, and it is possible that recall bias could confound our findings, wherein women were more likely to remember their provider discussing and/or encouraging them to have supplemental screening in situations where they went on to have the test. Finally, duration of the law may be associated with outcomes. While we required DBN states in our analysis to have been in place for at least 18 months at the time of survey administration, the three states with DBN‐coverage laws (CT, NJ, NY) had been in place longer (average 67 months) compared to states with generic DBN (average 41 months) and DBN + SS (average 46 months).

## 5 CONCLUSION

Our findings suggest that patient–provider discussions about and utilization of supplemental screening is significantly more common for women living in states with DBN‐coverage requirements. However, there was no evidence that other types of DBN laws were associated with supplemental screening behavior. Moreover, the overall level of knowledge regarding the potential risks of supplemental screening is sub‐optimal, as is the frequency with which patients are discussing these issues with their physicians. This is particularly important given the limited evidence base to support supplemental screening. Thus, in addition to legislative efforts to improve awareness and access to specific screening tests, additional evidence regarding effectiveness as well as provider education on enhancing shared decision in the context of clinical uncertainty is warranted.

## CONFLICT OF INTEREST

Dr Gross has received research funding through Yale from Johnson & Johnson and an NCCN/Pfizer program. In addition, Dr Gross has received compensation from Flatiron Health for travel and speaking. Dr. Richman was supported by 1 K08 CA248725‐01A1. The other authors declare that they have no conflict of interest.

## AUTHORS CONTRIBUTIONS

Jenerius A. Aminawung: Study conception and design, data analysis, writing – original draft, and writing – review and editing. Jessica R. Hoag: Study conception and design, data analysis, writing – original draft, and writing – review and editing. Kelly A. Kyanko: Writing – review and editing. Xiao Xu: Writing – review and editing. Ilana B. Richman: Writing – review and editing. Susan H. Busch: Writing – review and editing. Cary P. Gross: Study conception and design, funding acquisition, and writing – review and editing.

## Supporting information

Supplementary Material

## Data Availability

The dataset analyzed during the current study is available from the senior author, Dr Gross, on reasonable request.
