# Supplementary material for: Breast cancer supplemental screening: Women’s knowledge and utilization in the era of dense breast legislation
Source: Cancer Med. 2020 Jun 14;9(15):5662–71. doi: 10.1002/cam4.3218 (PMC7402830; doi:10.1002/cam4.3218)
Supplement: Supplementary file 1 — Supplementary Material [file CAM4-9-5662-s001.docx]

Base: All respondents

S1 [S]

Have you ever been diagnosed with breast cancer?

1. No

2. Yes

*IF S1=2 (YES) OR REFUSED, TERMINATE AND INSERT STANDARD CLOSE.*

Base: Qualified respondents

S2 [S]

***Screening*** *is used to look for spots or suspicious findings in women who do not have any worrisome signs or symptoms of breast cancer.*

***Mammogram***

*A mammogram is a routine screening exam that takes x-rays of the breasts by a machine that presses against the breast. Screening mammograms play a role in early breast cancer detection in women who have no signs or symptoms of breast cancer.*

*[INSERT Mammogram.jpg]*

In the past 18 months, have you had a routine screening mammogram?

1. No

2. Yes

3. Don’t know

Base: S2=1, 3 or refused

S3 [S]

Have you ever had a routine screening mammogram?

1. No

2. Yes

Base: S3=1 (No) or refused

Script: Randomize

S4 [M]

You said that you have never had a routine screening mammogram. What is the reason you did not have a mammogram?

1. Did not think it was necessary/it was not necessary
2. Couldn’t afford the test (out-of-pocket costs)
3. Too painful, unpleasant, or embarrassing
4. Couldn’t get time off of work/Didn’t have time
5. Problems with transportation
6. Didn’t know where to get the test
7. I’m too young
8. I don’t have a doctor
9. Anxiety/fear
10. No reason/never thought about it **[S][Anchor]**
11. Other **[Specify] [O] [Anchor]**

*IF S3=1 OR REFUSED, TERMINATE AND INSERT STANDARD CLOSE.*

Base: S3=2 (Yes)

S5 [S]

How long has it been since you had your last routine screening mammogram?

1. Within the past 2 years (more than 18 months but less than 2 years ago)
2. Within the past 3 years (more than 2 years but less than 3 years ago)
3. Within the past 5 years (more than 3 years but less than 5 years ago)
4. 5 or more years ago

Base: S3=2 (Yes)

Script: Randomize

S6 [M]

What is the reason you did not have a mammogram in the past 18 months?

1. Did not think it was necessary/it was not necessary
2. Couldn’t afford the test (out-of-pocket costs)
3. Too painful, unpleasant, or embarrassing
4. Couldn’t get time off of work/Didn’t have time
5. Problems with transportation
6. Didn’t know where to get the test
7. I’m too young
8. I don’t have a doctor
9. Anxiety/fear
10. No reason/never thought about it **[S][Anchor]**
11. Other **[Specify] [O][Anchor]**

Base: Qualified respondents

S7 [S] [PROMPT]

*Breast density is measured on a mammogram and is the amount of connective tissue compared to fatty tissue. Dense breast tissue has less fat and more connective tissue, which appears white on a mammogram. About 2/3 of women before menopause and 1/4 of women after menopause have dense breast tissue.*

Have you ever been informed by a health care provider and/or mammogram letter that you have dense breasts?

1. No

2. Yes

Base: S7=2 (Dense breasts)

S8 [S]

You said that you have been informed by a health care provider or mammogram letter that you have dense breasts. How long has it been since you were told you had dense breasts?

1. Within the past 18 months
2. Within the past 2 years (more than 18 months but less than 2 years ago)
3. Within the past 3 years (more than 2 years but less than 3 years ago)
4. Within the past 5 years (more than 3 years but less than 5 years ago)
5. 5 or more years ago

Base: S7=1 (Does not have dense breasts)

Q0 [S]

Before this survey, have you heard about breast density?

1. No

2. Yes

Base: Q0=2(Have heard about breast density)

Q1 [S]

Have you ever talked about your breast density with a health care provider such as a doctor, nurse practitioner, or physician assistant?

1. No

2. Yes

Base: Q1=2 (talked to HC provider about breast density)

Q2 [S]

Who brought up your most recent discussion about breast density?

1. I brought up the topic of breast density with my health care provider

2. My health care provider brought up the topic of breast density with me

Base: Qualified respondents

Q3 [S]

Where did you receive your most recent routine screening mammogram?

1. Hospital setting (radiology department)

2. Radiology or imaging center not in a hospital

3. Mammography van or mobile mammography unit

4. Specialty breast clinic (that offers only breast care and mammograms)

5. Other [Specify] [O]

Base: Qualified respondents

Q4 [S Horizontal]

If a woman has dense breasts, how does this affect the ability of a mammogram to find breast cancer?

1. Makes it much easier
2. Makes it a little easier
3. Does not impact
4. Makes it a little more difficult
5. Makes it much more difficult
6. Don’t know

Base: Qualified respondents

Q5 [S Horizontal]

If a woman has dense breasts, how does this affect the chance of her getting breast cancer at some point in her life?

1. Large decrease in chance
2. Small decrease in chance
3. Does not influence chance
4. Small increase in chance
5. Large increase in chance
6. Don’t know

Base: Qualified respondents

Q6 [S]

How do you perceive or think about dense breasts?

1. Dense breasts are normal
2. Dense breasts are a disease
3. Dense breasts are an abnormal finding on a mammogram

Base: Qualified respondents

[DISPLAY1]

**DID YOU KNOW?**

**Women with dense breasts may be offered one of the following three options for screening**

**Screening** is used to look for spots or suspicious findings in women who do not have any worrisome

signs or symptoms of breast cancer.

**Screening Option 1: Mammogram only**

**In this option, women with dense breasts get a mammogram only**

*A mammogram takes x-rays using a machine that presses up against the breast.*

*[INSERT Mammogram.jpg]*

**Screening Option 2: Both Mammogram and Breast MRI**

**In this option, women with dense breasts get an MRI test, *even if* the mammogram doesn’t show any suspicious findings.**

*Breast magnetic resonance imaging (MRI) is a screening strategy that uses magnetic fields to create an image of the breast.*

+

*[INSERT Mammogram.jpg] [INSERT MRI.jpg]*

**Screening Option 3: Both Mammogram and Breast Ultrasound**

**In this option, women with dense breasts get an ultrasound exam, *even if* the mammogram doesn’t show any suspicious findings**

*Breast ultrasound uses sound waves to make a computer picture of the breast.*

+

*[INSERT Mammogram.jpg] [INSERT breast-ultrasound.jpg]*

Base: Qualified respondents

[DISPLAY2]

***If a screening option finds a spot or suspicious finding, a woman might have more tests to diagnose the issue. Here is a description of the difference between screening tests and diagnostic tests:***

| ***SCREENING*** | ***DIAGNOSTIC*** |
| --- | --- |
| Look for spots or suspicious findings in women who do not have any worrisome signs or symptoms of breast cancer | If a screening test finds something suspicious, a woman will get extra tests. These extra tests are meant to diagnose the suspicious finding and find out if it’s cancer (these are called diagnostic tests), and may include additional mammogram, additional MRI, additional ultrasound, or biopsy. |

*[INSERT Screening and diagnostic tests.jpg]*

Base: Qualified respondents

Q7a [S]

**Research has shown that if 1,000 women age 45 with dense breasts get option1; the “mammograms only” screening option, each year for 10 years, about 1-2 deaths will be prevented.**

*[INSERToption1_display3_NEW.jpg]*

**Now let us consider what would happen if 1,000 women age 45 with dense breasts get option2; the “mammograms + MRI” screening option, each year for 10 years, compared to 1,000 women that get Option 1: “mammogram alone”.**

*[INSERT* *option2_deaths_NEW.jpg]*

Would you say adding MRI to mammogram will …

(If you don’t know, make your best guess)

1. Prevent many more deaths compared to mammogram alone

2. Prevent slightly more deaths compared to mammogram alone

3. Prevent the same number of deaths compared to mammogram alone

4. Lead to slightly more deaths compared to mammogram alone

5. Lead to many more deaths compared to mammogram alone

Base: Qualified respondents

Q7b [S]

*[INSERToption1_display3_NEW.jpg]*

How do you think women age 45 with dense breasts who receive **Screening Option3: “Mammogram + Ultrasound”** each year, compare to Screening Option1: “Mammogram only” in terms of the number of deaths prevented? If you don’t know, make your best guess.

*[INSERT option3_deaths_NEW.jpg]*

Would you say adding Ultrasound to mammogram will …

(If you don’t know, make your best guess)

1. Prevent many more deaths compared to mammogram alone

2. Prevent slightly more deaths compared to mammogram alone

3. Prevent the same number of deaths compared to mammogram alone

4. Lead to slightly more deaths compared to mammogram alone

5. Lead to many more deaths compared to mammogram alone

Base: Qualified respondents

Q8a [S]

**Sometimes when screening shows a spot or suspicious finding, a needle biopsy is needed. A needle biopsy uses a hollow needle to remove samples of tissue from the breast so they can be studied in the lab to see if cancer is present.**

**Research has shown that if 1,000 women age 45 with dense breasts start having the “mammograms only” screening option each year for 10 years, about 100 women would have a spot or suspicious finding that requires a biopsy but turns out not to be cancer.**

*[INSERT* *option1_display4_NEW.jpg]*

How do you think women age 45 with dense breasts who receive **Screening Option2: “Mammogram + MRI”**, compare to Screening Option1: “Mammogram only” in terms of the number who would have a spot or suspicious finding that requires a biopsy but turns out not to be cancer? If you don’t know, make your best guess.

*[INSERT option2_biopsy_NEW.jpg]*

1. Many more biopsies required
2. Slightly more biopsies required
3. Same number of biopsies required
4. Slightly fewer biopsies required
5. Many fewer biopsies required

Base: Qualified respondents

Q8b [S]

*[INSERT option1_display4_NEW.jpg]*

How do you think women age 45 with dense breasts who receive **Screening Option3: “Mammogram + Ultrasound”** each year, compare to Screening Option1: “Mammogram only” in terms of the number who would have a spot or suspicious finding that requires a biopsy but turns out not to be cancer? If you don’t know, make your best guess.

*[INSERT(New image) option3_biopsy_NEW.jpg]*

1. Many more biopsies required
2. Slightly more biopsies required
3. Same number of biopsies required
4. Slightly fewer biopsies required
5. Many fewer biopsies required

Base: Qualified respondents

Q9 [S]

***Screening tests are different from diagnostic tests. Here is a description of the difference between screening tests and diagnostic tests:***

| ***SCREENING MRI*** | ***DIAGNOSTIC MRI*** |
| --- | --- |
| Look for spots or suspicious findings in women who do not have any worrisome signs or symptoms of breast cancer | If a screening test finds something suspicious, a woman will get extra tests. These extra tests are meant to diagnose the suspicious finding and find out if it’s cancer (these are called diagnostic tests). |

*[INSERT Mammo plus MRI screening_option2.jpg]*

Before this survey, have you heard of mammogram + MRI for screening?

1. No

2. Yes

Base: Q9=2 (Have heard of mammogram + MRI screening)

Q10 [M]

In the past 18 months, which of the following have you discussed with your health care provider about breast MRI for screening?

1. I have not discussed breast MRI with provider [S]
2. The risks
3. The benefits
4. The costs
5. We discussed other aspects of breast MRI, aside from risks, benefits or costs.

Base: Q9=2 (Have heard of mammogram + MRI screening)

Q11 [S]

In the past 18 months, have you been encouraged to have a breast MRI for screening by your health care provider?

1. No

2. Yes

Base: Q9=2 (Have heard of mammogram + MRI screening)

Q12 [M]

In the past 18 months, have you had a breast MRI?

1. No [S]
2. Yes, because my mammogram found a spot or suspicious finding
3. Yes, because my mammogram results said I had dense breasts
4. Yes, because of other reasons ( family history, my genetic risk)
5. Yes, but I don’t know why [S]

Base: Q12=2-4 (Have had a breast MRI)

Q13 [S]

You indicated that you had a breast MRI for screening in the past 18 months. Approximately how much of your own money did you pay (i.e. out-of-pocket) for this test?

1. $0
2. $1-24
3. $25-49
4. $50-99
5. $100-299
6. $300 or more
7. Don’t Know

Base: Q12=2-4 (Have had a breast MRI)

Q14 [S]

What did you think about the out-of-pocket cost of the breast MRI for screening?

1. Less than what I expected to pay
2. Same as what I expected to pay
3. More than what I expected to pay
4. Don’t Know

Base: Q12=1 (Have not had a mammogram + MRI screening)

Scripter: Randomize

Q15a [M]

Which of the following best describe the reasons you delayed or did not have the breast MRI?

1. Did not think it was necessary/it was not necessary
2. Couldn’t afford the test (out-of-pocket costs)
3. Too painful, unpleasant, or embarrassing
4. Couldn’t get time off of work/Didn’t have time
5. Problems with transportation
6. Didn’t know where to get the test
7. I’m too young
8. I don’t have a doctor
9. Anxiety/fear
10. No reason/never thought about it [s]
11. Other [Specify]

Base: Q15a=1-11

Script: If only one answer provided in A15a please auto-fill

Q15b [S]

Of the reasons you selected in the previous question, what would you say is the one most important reason you delayed or did not have the breast MRI?

1. [IF Q15a_1=1] No reason/never thought about it
2. [IF Q15a_2=1] Did not think it was necessary/it was not necessary
3. [IF Q15a_3=1] Couldn’t afford the test (out-of-pocket costs)
4. [IF Q15a_4=1] Too painful, unpleasant, or embarrassing
5. [IF Q15a_5=1] Couldn’t get time off of work/Didn’t have time
6. [IF Q15a_6=1] Problems with transportation
7. [IF Q15a_7=1] Didn’t know where to get the test
8. [IF Q15a_8=1] I’m too young
9. [IF Q15a_9=1] I don’t have a doctor
10. [IF Q15a_10=1] Anxiety/fear
11. [IF Q15a_11=1] **[Insert answer from Q15a=11]**

Base: Qualified respondents

Q16 [S]

***Screening tests are different from diagnostic tests. Here is a description of the difference between screening tests and diagnostic tests:***

| ***SCREENING Ultrasound*** | ***DIAGNOSTIC Ultrasound*** |
| --- | --- |
| Look for spots or suspicious findings in women who do not have any worrisome signs or symptoms of breast cancer | If a screening test finds something suspicious, a woman will get extra tests. These extra tests are meant to diagnose the suspicious finding and find out if it’s cancer (these are called diagnostic tests). |

Before this survey, have you heard of mammogram + ultrasound for screening?

*[INSERT Mammo plus US screening_option3.jpg]*

1. No
2. Yes

Base: Q16=2 (Heard of mammogram + ultrasound for screening)

Q17 [M]

In the past 18 months, which of the following have you discussed with your health care provider about breast ultrasound for screening?

1. I have not discussed breast ultrasound with provider [s]
2. The risks
3. The benefits
4. The costs
5. We discussed other aspects of breast ultrasound, aside from risks, benefits or costs

Base: Q16=2 (Heard of mammogram + ultrasound for screening)

Q18 [S]

In the past 18 months, have you been encouraged to have a breast ultrasound for screening by your health care provider?

1. No
2. Yes

Base: Q16=2 (Heard of mammogram + ultrasound for screening)

Q19 [M]

In the past 18 months, have you had a breast ultrasound?

1. No [S]
2. Yes, because my mammogram found a spot or suspicious finding
3. Yes, because my mammogram results said I had dense breasts
4. Yes, but I don’t know why [S]

Base: Q19=2-4 (Have had breast ultrasound)

Q20 [S]

You indicated that you had a breast ultrasound for screening in the past 18 months. Approximately how much of your own money did you pay (i.e. out-of-pocket) for this test?

1. $0
2. $1-24
3. $25-49
4. $50-99
5. $100-299
6. $300 or more
7. Don’t Know

Base: Q19=2-4 (Have had breast ultrasound)

Q21 [S]

What did you think about the out-of-pocket cost of the breast ultrasound for screening?

1. Less than what I expected to pay
2. Same as what I expected to pay
3. More than what I expected to pay
4. Don’t Know

Base: Q19=1 (Have not had breast ultrasound)

Q22a [M]

Which of the following best describe the reasons you delayed or did not have the breast ultrasound?

1. Did not think it was necessary/it was not necessary
2. Couldn’t afford the test (out-of-pocket costs)
3. Too painful, unpleasant, or embarrassing
4. Couldn’t get time off of work/Didn’t have time
5. Problems with transportation
6. Didn’t know where to get the test
7. I’m too young
8. I don’t have a doctor
9. Anxiety/fear
10. No reason/never thought about it [S]
11. Other [O]

Base: Q19=1 (Have not had breast ultrasound)

Scripter: Auto punch if only 1 item selected in Q22a

Q22b [S]

Of the reasons you selected in the previous question, what would you say is the one most important reason you delayed or did not have the breast ultrasound?

1. [IF Q22a_1=1] Did not think it was necessary/it was not necessary
2. [IF Q22a_1=2] Couldn’t afford the test (out-of-pocket costs)
3. [IF Q22a_1=3] Too painful, unpleasant, or embarrassing
4. [IF Q22a_1=4] Couldn’t get time off of work/Didn’t have time
5. [IF Q22a_1=5] Problems with transportation
6. [IF Q22a_1=6] Didn’t know where to get the test
7. [IF Q22a_1=7] I’m too young
8. [IF Q22a_1=8] I don’t have a doctor
9. [IF Q22a_1=9] Anxiety/fear
10. [IF Q22a_1=10] No reason/never thought about it [S]
11. [IF Q22a_1=111] **[Insert answer from Q22a=11]**

Base: Qualified respondents

Q23 [S]

Have you ever had a breast cancer screening test that showed a spot or finding that was suspicious for cancer, but turned out not to be breast cancer (a “false-positive”)?

1. No
2. Yes

Base: Q23=2 (Had breast cancer screening that showed a spot)

Q24 [S]

How long has it been since you had a breast cancer screening test that showed a spot or finding that was suspicious for cancer, but turned out not to be breast cancer (a “false-positive”)?

1. Within the past month
2. 1 to 3 months ago
3. 3 to 6 months ago
4. 6 to 12 months ago
5. 12 to 18 months ago
6. More than 18 months (year and half) ago

Base: All qualified respondents

Q25 [Grid, S per row]

Think of the one month in the past 18 months (year and a half) when you were most concerned about breast cancer or breast cancer screening/testing. During that month, how often did you experience the following because of thoughts and feelings about breast cancer?

Statements per row:

1. I have been worried about my future [because of thoughts and feelings about breast cancer]
2. I have felt scared [because of thoughts and feelings about breast cancer]
3. I have been upset [because of thoughts and feelings about breast cancer]
4. I have felt restless [because of thoughts and feelings about breast cancer]
5. I have been nervous [because of thoughts and feelings about breast cancer]
6. I have felt terrified [because of thoughts and feelings about breast cancer]

Statements per column:

1. Not at all
2. A bit
3. Quite a bit
4. A lot

Base: Qualified respondents

Q26 [S]

Some states have laws that require insurance to pay for screening MRI and ultrasound for women with dense breasts. Does [INSERT PPSTATEN NAME] have one of these laws?

1. No
2. Yes
3. Don’t Know

Base: Qualified respondents

QT1 [S]

*A 3-D mammogram, also called digital breast mammography with tomosynthesis, is a screening exam in which a machine takes many low-dose x-rays as it moves in an arc over the breast. The images taken can be combined into a 3-dimensional picture.*

*[INSERT Digital Breast Mammogram.jpg]*

Have you ever had a 3-D mammogram, also known as digital breast tomosynthesis?

1. No
2. Yes

***CREATE DOV_ASSIGN1 [S]***

***RANDOMLY ASSIGN A VALUE OF 1 TO 4***

***1=$10, 2=$50, 3=$100, 4=$200***

Base: Qualified respondents

QT2 [S]

Studies have shown that a 3-D mammogram finds more cancers than a regular mammogram, while at the same time lowers the number of false-positive findings.

For every 1,000 women screened with a 3-D mammogram compared to a regular mammogram:

- Approximately 1 more cancer will be found
- Approximately 15 fewer healthy women will have to come back for additional test.

Knowing this information, would you be willing to pay [INSERT DOV_ASSIGN1] of your own money out-of-pocket to receive a 3-D mammogram instead of a regular mammogram?

1. No
2. Yes

Base: QT2=1 (Not willing to pay proposed amount)

QT3a [S]

You said that you would not be willing to pay [INSERT DOV_ASSIGN1] of your own money out-of-pocket. [DOV_ASSIGN1=1: Would you be willing to receive the test at no coast to you? / DOV_ASSIGN1=2 Would you be willing to pay $10 / DOV_ASSIGN1=3: Would you be willing to pay $50 / DOV_ASSIGN1=4 Would you be willing to pay $100]?

1. No
2. Yes

Base: QT3a=1 (Not willing to pay proposed amount) and dov_assign1=3 or 4

QT3b [S]

You said that you would not be willing to pay [DOV_ASSIGN1=3: $50 / DOV_ASSIGN1=4: $100] of your own money out-of-pocket. Would you be willing to pay [DOV_ASSIGN1=3: $10 / DOV_ASSIGN1=4 $50]?

1. No
2. Yes

Base: QT3b=1 (Not willing to pay proposed amount) and dov_assign1=4

QT3c [S]

You said that you would not be willing to pay [DOV_ASSIGN1=4: $50] of your own money out-of-pocket. Would you be willing to pay [DOV_ASSIGN1=4 $10]?

1. No
2. Yes

Base: QT2=2 (Willing to pay proposed amount)

QT4a [S]

You said that you would be willing to pay [INSERT DOV_ASSIGN1] of your own money out-of-pocket. Would you be willing to pay [DOV_ASSIGN1=1: $50 / DOV_ASSIGN1=2 $100 / DOV_ASSIGN1=3: $200 / DOV_ASSIGN1=4 $500]?

1. No
2. Yes

Base: QT4a=2 (Willing to pay proposed amount) and dov_assign1=1-3

QT4b [S]

You said that you would be willing to pay [DOV_ASSIGN1=1: $50 / DOV_ASSIGN1=2 $100 / DOV_ASSIGN1=3: $200] of your own money out-of-pocket. Would you be willing to pay [DOV_ASSIGN1=1: $100 / DOV_ASSIGN1=2 $200 / DOV_ASSIGN1=3: $500]?

1. No
2. Yes

Base: QT4b=2 (Willing to pay proposed amount) and dov_assign1=1-2

QT4c [S]

You said that you would be willing to pay [DOV_ASSIGN1=1: $100 / DOV_ASSIGN1=2: $200] of your own money out-of-pocket. Would you be willing to pay [DOV_ASSIGN1=1: $200 / DOV_ASSIGN1=2 $500]?

1. No
2. Yes

Base: QT4c=2 (Willing to pay proposed amount) and dov_assign1=1

QT4d [S]

You said that you would be willing to pay [DOV_ASSIGN1=1: $200] of your own money out-of-pocket. Would you be willing to pay [DOV_ASSIGN1=1: $500]?

1. No
2. Yes

Base: Qualified respondents

QK1

*Mammograms can help to detect breast cancer at an early stage, and treatment of early stage cancer can prevent serious illness or death. However, some breast cancers that are found on a mammogram will not cause symptoms or any other health problems in a woman’s lifetime. This might happen because the cancer never grows or spreads, or when the women die from another disease before the breast cancer had a chance to cause any health problems.*

*Have you ever heard about this idea that some breast cancers might be detected with screening, but will not cause symptoms or any other health problems in a woman’s lifetime ?*

1. *No*
2. *Yes*
3. *I don’t know*

Base: Qualified respondents

QK2 [S]

The idea that some breast cancers might be detected with screening but *will not cause symptoms or any other health problems in a woman’s lifetime is called “overdiagnosis”.*

How much do you agree or disagree with the following statements?

The idea of overdiagnosis influences my decision when considering whether to get a mammogram

Would you say…

1. Strongly disagree
2. Disagree
3. Neither agree nor disagree
4. Agree
5. Strongly agree

The idea of overdiagnosis influences my decision when considering whether to get additional screening tests such as a breast MRI or a screening Ultrasound

Would you say …

1. Strongly disagree
2. Disagree
3. Neither agree nor disagree
4. Agree
5. Strongly agree

Base: Qualified respondents

QK3 [S]

How frequently do you usually get a screening mammogram?

1. Every year
2. Every 2 years
3. Every 3 years
4. Other ______________

Base: If QK3 =1 (Every year)

QK4 [S]

Some guidelines suggest that women can opt to have their mammograms every two years, rather than every year, in some instances.

Are you aware that for some women, getting a mammogram every two years is an acceptable screening strategy?

1. No
2. Yes
3. I don’t know

Base: If QK3 =1 (Every year)

QK5 [S]

If your doctor suggested that you have your mammogram every two years, what would you do?

1. Start getting my mammograms every 2 years
2. Continue getting mammograms every year
3. Get mammograms at some other time interval (i.e. neither every year nor every 2 years)
4. I don’t know

Base: If QK5=2 (Continue getting mammograms every year)

QK6 [M]What is the reason you would have mammograms every year even if your doctor suggested that you could have a mammogram every two years?

1. Yearly mammograms are better at detecting breast cancer
2. Getting yearly mammograms puts my mind at ease
3. Annual mammograms are part of my regular health routine, and I wouldn’t want to change that
4. Other **______________________________________**

[INSERT STANDARD CLOSE]

END OF QUESTIONNAIRE
